# Bone ultrasound velocity in pediatric intensive care unit: a pilot study

**DOI:** 10.1186/2036-7902-5-8

**Published:** 2013-10-31

**Authors:** Ayelet Zerem, Francis B Mimouni, Elie Picard, Sarit Shahroor

**Affiliations:** 1 Pediatric Intensive Care Unit, Shaare Zedek Medical Center, affiliated to The Hebrew University School of Medicine and Pediatric department, Jerusalem 91031, Israel; 2 Pediatric Pulmonary Unit, Shaare Zedek Medical Center, affiliated to The Hebrew University School of Medicine and Pediatric department, Jerusalem 91031, Israel; 3 Tel Aviv Medical Center, the Sackler School of Medicine, Tel Aviv 69978, Israel

**Keywords:** Bone speed of sound, Bone strength, Immobilization, Catabolic state, Paralysis

## Abstract

**Background:**

Bone loss has been documented in adults in intensive care wards. Children admitted to pediatric intensive care units (PICU) are also exposed to many potential risk factors for bone loss such as immobilization, catabolic state, and nutritional depletion. Quantitative ultrasound technique that measures speed of sound (SOS) correlates with bone mineral density (BMD) and strength. Herein is a clinical prospective longitudinal, observational pilot study to evaluate early bone changes that occur during the first few days of PICU admission.

**Methods:**

Children are hospitalized in a pediatric intensive under general anesthesia and muscle paralysis. Bone SOS at the mid-shaft tibia was measured on the first day of hospitalization and on days 2 to 3 thereafter.

**Results:**

Nineteen children were studied. Bone SOS decreased during the first 3 days of hospitalization from 3,297 ± 315 to 3,260 ± 311 m/min (*p* < 0.05). The decrease was approximately 1% of the original SOS over the first 2 to 3 days of admission.

**Conclusion:**

There is a significant decrease in bone strength after 3 days in pediatric patients admitted to an intensive care department. Longitudinal studies of a larger group of children are necessary to determine the clinical meaning of the results and to possibly evaluate preventive approaches.

## Background

Many studies in humans and in various animal models have demonstrated a decrease in bone mineral density (BMD) associated with immobilization. Indeed, whenever the skeleton is unloaded, because of continued bed rest, reduction in mechanical use or microgravity, a series of events occur, that result in a loss of bone mineral content. Subsequently, bone strength decreases, enhancing the risk of bone fractures. Hypercalcemia and kidney stones are likely related to these events [1-3].

Examples of such events may be found in the decreased bone density observed in healthy adults after a prolonged bed rest [4,5], or in healthy cosmonauts affected by microgravity [5]. Healthy volunteers exposed to single limb suspension have a significant bone loss in the suspended limb compared to its active match within 7 to 21 days [6]. Significant bone loss has also been documented in adult patients immobilized because of acute spinal cord injury [7], paralysis after stroke [8], and during an hospitalization in an intensive care ward [9,10]. A study that examined 49 ventilator-dependent chronically critically ill patients found an increase in metabolic markers of bone resorption in 92% of the patients [11].

Several studies performed in children have also demonstrated the effect of immobilization upon bone mass. For instance, decreased bone mass has been shown to be present in children suffering from cerebral palsy or chronic neuromuscular disorders [12-14]. Bone loss is also a well-known complication of severe burns [14] and of orthopedic injuries [15]. In children after fractures, a decrease in bone mass has been demonstrated in the injured limb [16].

Little is known about the bone changes that occur in children admitted to a pediatric intensive care unit. Theoretically, these children are exposed to many risk factors for bone loss. Among them: immobilization (due to neurologic condition, sedation, or paralysis), catabolic state and endocrine imbalance, nutritional depletion, sun deprivation, and at times, use of medications that promote bone loss such as steroids, antiepileptic drugs [17], diuretics [18], or heparin [19].

Quantitative ultrasound (QUS) method provides an acceptable non-invasive alternative to assess bone mass [20,21]. The bone speed of sound (SOS) is in correlation with bone mineral density and even more with cortical thickness and bone elasticity [22]. Thus it is an acceptable estimate of bone strength. We therefore designed this prospective, longitudinal observational study to evaluate the early bone changes (SOS) that occur during the first few days of hospitalization in the pediatric intensive care unit (PICU). We hypothesized that SOS decreases significantly within the first few days of admission in critically ill PICU patients.

## Methods

The study was designed to be a pilot, observational one that would enable us to perform later on sample size calculations for a larger study, if needed.

Patients admitted to the PICU of the Shaare Zedek Medical Center between September 2008 and June 2009 were prospectively enrolled in the study. We only included patients that required sedation and ventilation for their clinical management. Patients were excluded if they had any kind of previously known inherited or acquired bone disease. Children involved with any kind of trauma with or without bone fractures were also excluded. The study was approved by the local institutional Helsinki committee, and all the parents' participants signed a written consent.

Bone SOS was measured using the Sunlight Omnisense 7000S quantitative ultrasound bone sonometer device (Sunlight Ltd., Tel Aviv, Israel), and results were expressed as SOS (m/s). Bone SOS is usually regarded more a measurement of bone strength than a measurement of bone mineral density. Measurements were performed at the inner part of the mid-shaft of the tibia. In order to reduce inter-observer variability, the same operator (AZ) performed all examinations. Bone SOS was measured within the first 24 h of admission, and once again at 48 to 72 h after admission on the same side. The instrument averages three statistically consistent measurements. In case five measurements do not produce three close results, the procedure is aborted automatically by the instrument and has to be repeated. In our hands, the coefficient of variation of this method after repositioning is below 0.5%.

In all participants, selected relevant demographic and clinical data were prospectively collected and recorded, including acute and or chronic medical conditions, medical treatment, and nutritional evaluation.

Statistical analyses: the Minitab version 15.0 (State College, PA, USA) was used for statistical analyses. We used paired Student's *t* test to determine whether the change between the SOS in admission and during hospitalization in PICU was significant. Results are expressed as mean ± 1SD. A *P* value of <0.05 was considered significant.

## Results

A total of 19 children were recruited in the study. Table 1 depicts selected major demographic characteristics of the patients including age, gender, diagnosis in admission to PICU, chronic illnesses, and medications received during their stay that might potentially affect bone density.

**Table 1:** Selected demographic and clinical patient characteristics

**Patient**	**Age (years)**	**Gender**	**Diagnosis**	**Chronic diseases**	**Medications**
1	15	F	Retropharyngeal mass	None	1,3,4
2	10	M	Acute myoglobinuria	None	1,4
3	0.04	F	Bilateral pyeloplasty	UPJ stenosis	1,4
4	7	M	Pneumococcal sepsis, coma	Trisomy 21	1,3
5	2	M	Tracheitis	None	1,4
6	1	F	Lymphadenitis	None	1,3,4
7	2	F	Respiratory distress	Prematurity, BPD.PMR*	1,3,4
8	0.5	M	T-E fistula repair	T-E fistula	1,4
9	1.5	F	Pleuropneumonia	None	1,4
10	0.3	M	Near SIDS, coma	None	1,3,4
11	1.5	F	Caustic ingestion	None	1,3,4
12	0.1	M	Cardiogenic shock, infection	Congenital heart disease	1,3,4
13	1	F	Metabolic crisis first episode	SCHAD deficiency	1,4
14	0.3	M	Rotavirus enteropathy	None	1,4
15	0.5	F	Metabolic crisis first episode	GSD type 1	1,4
16	16	M	Stomach perforation	Severe PMR, gastrostomy	1,2
17	13	F	Perforated appendicitis	None	1,3
18	0.9	M	Right hemicolectomy	Prematurity, BPD	1
19	6	M	Respiratory failure	Multisystem autoimmune disorder	1,3

UPJ, ureteropelvic junction; BPD, bronchopulmonary dysplasia; PMR, psychomotor retardation; T-E, tracheoesophageal; SIDS, Sudden Infant Death Syndrome; SCHAD, short-chain hydroxyacyl-CoA dehydrogenase; GSD, glycogen storage disease. Medications: 1 - heparin, 2 - anticonvulsants, 3 - steroids, and 4 - furosemide.

Bone SOS decreased during hospitalization from 3,297 ± 315 to 3,260 ± 311 m/min, which is a difference of 37 ± 73 m/min, or approximately 1.1% of the original SOS. By paired *t* test, this decrease in SOS was statistically significant (*p* < 0.05).

## Discussion

In this pilot study of 19 critically ill children admitted to a PICU, ventilated and sedated, we observed a small but statistically significant decrease in SOS (a little more than 1% of its initial value) within the relatively short period of 2 to 3 days. Since SOS has been shown to correlate with bone mineral density and even more with bone strength [22], we speculate that the decrease in SOS observed in our patients reflected a decrease in BMD as well as in bone strength.

Previous studies in adults' intensive care patients reported a decrease in bone density during hospitalization [9-11]. Moreover, in adults hospitalized in intensive care units, indices of bone resorption worsen as the hospitalization is prolonged [10]. While there are studies showing the effects of immobilization upon BMD and upon SOS in adults or children following orthopedic injuries [15,16], or in acute clinical situations such as severe burns [14], we are not aware of a study similar to ours that documented early and rapid changes in SOS following admission in a PICU in critically ill, ventilated, sedated, and paralyzed children.

In this pilot study, no follow-up of sufficient period in a sufficient number of children has been performed, thus a limitation of our study is that we do not know whether the rate of decrease in SOS remains constant or changes over time. In addition, we did not evaluate variables that may have impacted upon the rate of decrease in bone SOS such as vitamin D status.

The mechanism by which BMD decreases in intensive care patients is probably multi-factorial. Among the potential culprits are (1) immobilization, which can be extreme under anesthesia or sedation and induced muscle paralysis; (2) metabolic and endocrine disturbances with secretion of stress hormones highly catabolic for bone such as cortisol; (3) use of medications such as steroids, heparin, furosemide, or anticonvulsants; (4) nutritional depletions in patients fed parenterally or unfed at all; and (5) sun deprivation. In our small number of patients, it was not possible to determine the relative effect of each and every one of these factors, and exposure to various bone-harming medications could only be mentioned in the table. All patients were in extreme hemodynamic and metabolic instability. Likewise, they were all treated in the first few days of their PICU admission with parenteral fluids without other type of nutritional support.

## Conclusions

Our pilot study demonstrates that bone SOS decreases significantly already over the first 2 to 3 days of admission of a child in a PICU. The clinical significance of such a decrease is unclear. We speculate that more prolonged immobilization, such as that occurring in ventilated patients may have even more prominent effects on bone metabolism. The mechanisms of the decrease in SOS, and the way by which it can be prevented, for instance by physical therapy [23] or by other physical approaches for prevention of bone loss [24] can only be explored in larger prospective trials.

## Competing interests

The authors declare that they have no competing interests.

## Authors' contributions

AZ has performed all the ultrasound studies, FBM did the statistical analysis, EP and SS wrote and reviewed the manuscript. All authors read and approved the final manuscript.
